# The Genetic Etiology of Tourette Syndrome: Large-Scale Collaborative Efforts on the Precipice of Discovery

**DOI:** 10.3389/fnins.2016.00351

**Published:** 2016-08-03

**Authors:** Marianthi Georgitsi, A. Jeremy Willsey, Carol A. Mathews, Matthew State, Jeremiah M. Scharf, Peristera Paschou

**Affiliations:** ^1^Department of Molecular Biology and Genetics, Democritus University of Thrace Alexandroupoli, Greece; ^2^Department of Medicine, Aristotle University of Thessaloniki Thessaloniki, Greece; ^3^Department of Psychiatry, University of California, San Francisco San Francisco, CA, USA; ^4^Department of Psychiatry, University of Florida School of Medicine Gainesville, FL, USA; ^5^Departments of Neurology and Psychiatry, Massachusetts General Hospital, Harvard Medical School Boston, MA, USA

**Keywords:** Gilles de la Tourette syndrome, genetics of complex disorders, neurodevelopmental disorders, GWAS (genomewide association study), gene-environment interactions, next generation sequencing, collaborative studies

## Abstract

Gilles de la Tourette Syndrome (TS) is a childhood-onset neurodevelopmental disorder that is characterized by multiple motor and phonic tics. It has a complex etiology with multiple genes likely interacting with environmental factors to lead to the onset of symptoms. The genetic basis of the disorder remains elusive. However, multiple resources and large-scale projects are coming together, launching a new era in the field and bringing us on the verge of discovery. The large-scale efforts outlined in this report are complementary and represent a range of different approaches to the study of disorders with complex inheritance. The Tourette Syndrome Association International Consortium for Genetics (TSAICG) has focused on large families, parent-proband trios and cases for large case-control designs such as genomewide association studies (GWAS), copy number variation (CNV) scans, and exome/genome sequencing. TIC Genetics targets rare, large effect size mutations in simplex trios, and multigenerational families. The European Multicentre Tics in Children Study (EMTICS) seeks to elucidate gene-environment interactions including the involvement of infection and immune mechanisms in TS etiology. Finally, TS-EUROTRAIN, a Marie Curie Initial Training Network, aims to act as a platform to unify large-scale projects in the field and to educate the next generation of experts. Importantly, these complementary large-scale efforts are joining forces to uncover the full range of genetic variation and environmental risk factors for TS, holding great promise for identifying definitive TS susceptibility genes and shedding light into the complex pathophysiology of this disorder.

## Introduction

Gilles de la Tourette Syndrome (TS; OMIM #137580) is a childhood-onset neurodevelopmental disorder, characterized by motor and vocal tics. Previous prevalence estimates ranged from 0.4 to 3.8% (Robertson, 2008); however, a recent meta-analysis refined the prevalence estimate to 0.3–0.9% (Scharf et al., 2015). TS often presents with co-morbidities such as attention deficit hyperactivity disorder (ADHD) and obsessive-compulsive disorder (OCD) (Swain et al., 2007), but autism spectrum disorders (ASD), depressive, and anxiety disorders may be also present (Hirschtritt et al., 2015). This overlap across disorders supports the hypothesis of a shared neurological background and genetic susceptibility (Mathews and Grados, 2011; Yu et al., 2015). Twin and family studies have long established that TS bears a strong genetic component (Pauls et al., 2014). However, TS is a complex disorder and has been associated with several environmental factors as well, with Group-A Streptococcal (GAS) infection and psychosocial stress being the most prominent among them (Hoekstra et al., 2013; Mathews et al., 2014). Despite the extensive research to unravel the genetic basis of TS, the field is still in its nascence. A simple PubMed search for “tic disorders” (26/3/2016) yields 5200 articles, far behind in comparison to those found for other childhood-onset neurodevelopmental disorders, such as ADHD (27,706 articles) and autism (33,167 articles), or related disorders such as OCD (16,445 articles). However, as presented at the First World Congress on Tourette Syndrome and Tic Disorders (London, June 24–26, 2015), and as described further here, the field of TS genetics stands at the precipice of discovery, thanks to the concerted efforts of multiple researchers from around the world and the coordination of multiple large-scale collaborative projects funded by the European Commission and the US National Institutes of Health.

The large-scale efforts outlined in this report, are complementary and represent a range of different approaches for the study of multifactorial disorders. The Tourette Syndrome Association International Consortium for Genetics (TSAICG) focuses on large families, sibpairs, trios, and cases for large case-control designs such as genomewide association studies (GWAS), copy number variation (CNV) scans, and exome/genome sequencing. TIC Genetics studies simplex trios as well as multigenerational families targeting rare, large effect size mutations. The European Multicentre Tics in Children Study (EMTICS) seeks to elucidate gene-environment interactions including the involvement of infection and immune mechanisms in TS etiology. Finally, TS-EUROTRAIN is a training network, aiming to act as a platform to unify large-scale projects in the field and educate the next generation of experts. To set the stage for the description of the aims of these consortia we briefly report the most notable findings that have shaped our current knowledge for TS genetic susceptibility (excellent exhaustive reviews are available in the literature) (State, 2011; Deng et al., 2012; Paschou, 2013; Sun et al., 2016).

### Candidate gene association studies

Based on findings from pathophysiological studies, hypotheses about the neuroanatomical regions affected in TS, and therapeutic response to neuroleptics, the first TS candidate genes were members of the dopaminergic, serotonergic, and glutamatergic pathways (Peterson et al., 2003; Kalanithi et al., 2005; Hartmann and Worbe, 2013; Table 1). Despite years of effort, results have been inconsistent, possibly owing to the small sample size of each individual study, the restricted number of variants explored in each study, and the inherent difficulties of candidate gene studies in genetically heterogenous disorders.

**Table 1:** **List of genes that have so far been implicated in TS etiology**.

**Experimental approach by which the gene was initially identified**	**Gene**	**Protein function**	**Study sample size**	**References**
**CANDIDATE GENE**				
Dopamine receptor D2	*DRD2*	Dopamine receptor	147 TS cases/314 controls	Comings et al., 1991
			225 TS cases/67 controls	Comings et al., 1996
			151 TS cases/183 controls	Lee et al., 2005
			69 trios	Herzberg et al., 2010
Dopamine receptor D4	*DRD4*	Dopamine receptor	12 trios/3 large families	Grice et al., 1996
			61 OCD cases with and without tics	Cruz et al., 1997
			110 trios	Díaz-Anzaldúa et al., 2004
Dopamine transporter [Solute carrier family 6 (neurotransmitter transporter), member 3]	*DAT1 (SLC6A3)*	Dopamine transporter	225 TS cases/67 controls	Comings et al., 1996
			103 trios	Tarnok et al., 2007
			266 TS cases/236 controls	Yoon et al., 2007
			110 trios	Díaz-Anzaldúa et al., 2004
Dopamine beta hydroxylase	*DBH*	Dopamine metabolism (transformation of dopamine to norepinephrine)	352 TS cases/148 controls	Comings et al., 1996
Monoamine oxidase-A	*MAOA*	Dopamine and serotonin metabolism (inactivation)	229 TS cases/90 relatives of TS/57 controls	Gade et al., 1998
			110 trios	Díaz-Anzaldúa et al., 2004
Serotonin receptor 1A	*HTR1A*	Serotonin receptor	56 TS cases/20 controls	Lam et al., 1996
Serotonin receptor 2C	*HTR2C*	Serotonin receptor	87 TS cases/311 controls	Dehning et al., 2010
Serotonin transporter [Solute carrier family 6 (neurotransmitter transporter), member 4]	*SERT (SLC6A4)*	Serotonin transporter	151 TS cases/858 controls	Moya et al., 2013
	*5-HTTLPR* locus	–	151 TS cases/858 controls	Moya et al., 2013
Tryptophan hydroxylase	*TPH2*	Serotonin metabolism (synthesis)	98 TS cases/178 controls	Mössner et al., 2007
Glutamate transporter [Solute carrier family 1 (glial high affinity glutamate transporter), member 3]	*EAAT1* (*SLC1A3*)	Glutamate transporter	256 TS cases/224 controls	Adamczyk et al., 2011
**CHROMOSOMAL ABERRATIONS**				
Slit and Trk-like, Family Member 1	*SLITRK1*	Neurite outgrowth	175 TS cases/2148 controls	Abelson et al., 2005
			222 trios	Karagiannidis et al., 2012
Inner mitochondrial membrane protein 2L	*IMMP2L*	Targets proteins to the inner mitochondrial membrane (exact role unknown)	*De novo* duplication in one TS patient	Petek et al., 2001
			*De novo* duplication in one TS patient	Kroisel et al., 2001
			*De novo* translocation in one TS patient	Patel et al., 2011
Contactin associated protein-like 2	*CNTNAP2*	Cell-cell interaction (myelinated axon-glia junction) and membrane potential (interaction with voltage-activated potassium channel)	One family	Verkerk et al., 2003
			460 TS cases/1131 controls	Fernandez et al., 2012
Neuroligin 4	*NLGN4*	Post-synaptic cell adhesion (synaptogenesis and synapse remodeling)	One family	Lawson-Yuen et al., 2008
**COPY NUMBER VARIANTS**				
Neurexin 1	*NRXN1*	Pre-synaptic cell adhesion (synapse formation)	111 TS cases/73 controls	Sundaram et al., 2010
			460 TS cases/1131 controls	Fernandez et al., 2012
			210 TS cases/285 controls	Nag et al., 2013
Arylacetamide deacetylase	*AADAC*	Detoxification (drug metabolism)—Unknown function in the brain	111 TS cases/73 controls	Sundaram et al., 2010;
			243 TS cases/1571 controls (initial study)	Bertelsen et al., 2015
			1181 TS cases/118730 controls (meta-analysis)	
Catenin alpha 3	*CTNNA3*	Cytoskeleton modeling (actin fillament assembly)	111 TS cases/73 controls	Sundaram et al., 2010
			460 TS cases/1131 controls	Fernandez et al., 2012
Fibrous sheath CABYR binding protein	*FSCB*	Ca2+-binding protein involved in fibrous sheath biogenesis	111 TS cases/73 controls	Sundaram et al., 2010
Voltage-gated potassium channel (*KCNE1, KCNE2*) and regulator of calcineurin 1 (*RCAN1*)	*KCNE1-KCNE2-RCAN1* locus	Neuronal cell membrane repolarization (*KCNE1, KCNE2*) and intracellular calcineurin-mediated signaling (*RCAN1*)	111 TS cases/73 controls	Sundaram et al., 2010
Collagen, type VIII, alpha 1	*COL8A1*	Connective tissue and basement membrane component (extracellular matrix collagen)	210 TS cases/285 controls	Nag et al., 2013
**LINKAGE STUDIES**				
Histidine decarboxylase	*HDC*	Histidine metabolism (synthesis)	One large family	Ercan-Sencicek et al., 2010
			520 trios	Karagiannidis et al., 2013
**GENOMEWIDE ASSOCIATION STUDIES**				
Collagen, type XXVII, alpha 1	*COL27A1* ^*^	Connective tissue component (extracellular matrix collagen)	1285 TS cases/4964 controls (initial GWAS)	Scharf et al., 2013
			1496 TS cases/5249 controls (meta-analysis)	
Netrin 4	rs2060546 (proximal to *NTN4*)	Extracellular protein that directs axon outgrowth and guidance	609 TS cases/610 controls (initial analysis)	Paschou et al., 2014
			1894 TS cases/5574 controls (meta-analysis)	

*As described in detail in the text, associations still remain inconclusive with difficulties in replicating original positive findings. To date, the heterogeneity of the disorder and small sample sizes have been hampering the identification of TS susceptibility genes and, large scale studies like the ones described in this perspective, hold the promise to unravel the genetic basis of TS*.

* *Only the top hit is shown here. No SNP reached genomewide significance levels*.

### Chromosomal aberration studies

*SLITRK1* has become the focus of debate in the TS literature following the discovery of a *de novo* inversion in a TS patient (Abelson et al., 2005). Although follow-up studies could not find novel *SLITRK1* mutations in a large number of patients (Deng et al., 2006; Chou et al., 2007; Scharf et al., 2008; Zimprich et al., 2008; Miranda et al., 2009), tagging-SNP-based association studies supported the implication of unidentified *SLITRK1* regulatory variants (Miranda et al., 2009; Karagiannidis et al., 2012). Tracing chromosomal aberrations in TS patients, *IMMP2L* has also been implicated in TS (Boghosian-Sell et al., 1996; Kroisel et al., 2001; Petek et al., 2001; Patel et al., 2011; Katuwawela, 2012) and other neurodevelopmental disorders, such as ASD, ADHD and dyslexia (Elia et al., 2010; Maestrini et al., 2010; Pagnamenta et al., 2010; Girirajan et al., 2011); yet *IMMP2L* coding mutations have not been identified (Petek et al., 2007). Other cytogenetic abnormalities associated with TS have implicated signal transduction and cell-adhesion proteins, such as CNTNAP2 (Verkerk et al., 2003; Poot et al., 2010) and NLGN4 (Lawson-Yuen et al., 2008; Table 1).

### CNV studies

Scans of structural variations in relation to TS have revealed *de novo* or recurrent rare CNVs in multiple genes (Table 1). Of particular interest is the significant overlap of rare CNVs observed in TS individuals with patients of other neuropsychiatric and neurodevelopmental disorders, including OCD, autism, ASD, and schizophrenia, suggesting shared etiology (Sundaram et al., 2010; Fernandez et al., 2012; McGrath et al., 2014).

### Linkage analysis studies

Early linkage studies on large multigenerational pedigrees failed to identify a major TS susceptibility gene (Deng et al., 2012; Paschou, 2013), and the single-gene hypothesis was soon abandoned. Recently, Ercan-Sencicek et al. identified an extremely rare non-sense mutation in *HDC*, in a unique family with several affected siblings, spurring again the interest for monogenic TS and introducing the involvement of the, until recently, ignored histaminergic pathway and its role in striatal dopamine regulation (Ercan-Sencicek et al., 2010; Castellan Baldan et al., 2014; Rapanelli et al., 2014). Although *HDC* mutations have been extremely rare in the literature (Lei et al., 2012), there is still evidence for association of the histaminergic pathway genes and TS (Fernandez et al., 2012; Karagiannidis et al., 2013).

### GWAS studies

The first TS GWAS was published in 2013, including 1285 cases and 4964 ancestry-matched controls. While no marker achieved a genomewide significance threshold, the strongest signal was observed for an intronic single nucleotide polymorphism (SNP) in *COL27A1* (Scharf et al., 2013). Moreover, a replication study of 42 top-signal SNPs from the first TS GWAS in 609 independent cases and 610 ancestry-matched controls, revealed the most significant association to date with a SNP lying closest to *NTN4*, an axon guidance molecule expressed in the developing striatum (Paschou et al., 2014). The first ever epigenome-wide association study of tic disorders revealed association signals nearby genes previously associated with neurological disorders that warrant further investigation (Zilhão et al., 2015).

## The tourette syndrome association international consortium for genetics (TSAICG)—genomewide association studies for TS

The TSAICG was founded in 1986 by TS genetic researchers in the United States and The Netherlands and brought together by the TSA-USA to exchange ideas and share preliminary data with the goal of identifying TS susceptibility genes. Early studies focused on parametric linkage analyses in large, multi-generational TS families (Pakstis et al., 1991; Barr et al., 1999) under the assumption that TS was a monogenic disorder. However, as evidence mounted to indicate the presence of non-Mendelian inheritance (Kurlan et al., 1994; Hasstedt et al., 1995), the TSAICG expanded to 11 clinical sites in USA, Canada, Germany, the UK, and the Netherlands to collect TS affected sibling pairs for non-parametric analyses using a standardized phenotypic assessment for TS, OCD, and ADHD, still used today by the three international TS consortia discussed here. The TSAICG was awarded NIH funding in 2000 to collect additional small nuclear families and completed a high-density linkage study of all existing affected sibpairs and multi-generational families (TSAICG, 2007). These analyses of over 2000 individuals identified a genomewide significant non-parametric linkage signal on chromosome 2p (TSAICG, 2007), though subsequent analyses have demonstrated significant heterogeneity across this locus, consistent with the presence of multiple distinct signals within the linkage region (O'Rourke et al., 2009). With the advent of the GWAS era, the TSAICG changed its collection goals to focus on association studies using both parent-proband trios and individual TS cases. These collections served as the basis for the first TS GWAS and parallel CNV analysis as described above (Scharf et al., 2013; McGrath et al., 2014). As it became clear that sample size is the major hindrance to gene discovery for complex neuropsychiatric traits, the TSAICG added additional recruitment sites and novel recruitment and assessment methods, such as web-based assessments of previously diagnosed TS cases and remote DNA collection using commercial laboratories across the US (Egan et al., 2012; Darrow et al., 2015). These online protocols facilitated collection of 1600 independent TS cases over the course of 2 years, a sample that served as the basis for the second TS GWAS and CNV studies whose preliminary results were presented at the First World Congress on Tourette Syndrome and Tic Disorders (to be published by fall 2016).

Each of these large-scale TS genetic studies have relied heavily on extended collaborations and data sharing, both within the TSAICG as well as across additional US and European research groups. The Gilles de la Tourette Syndrome GWAS Replication Initiative (GGRI) consists of multiple TS research groups across USA, Canada, France, Germany, Austria, Hungary, Italy, Greece and Poland, and formed out of an NIH TS Genetics Workshop following completion of the first TS GWAS. The GGRI collaborative resulted in both the targeted replication study described above (Paschou et al., 2014) and acted as another major contributing source for the second international TS GWAS and CNV studies. Similarly, TIC Genetics has contributed data from over 400 TS parent-proband trios to the latest TS GWAS. TSAICG and TIC Genetics are also currently collaborating in a joint analysis of exome sequencing data aimed at identifying recurrent, *de novo* mutations in TS parent-proband trio families (see below). Most recently, all of the above collaborative groups have also contributed their GWAS data to the Psychiatric Genomics Consortium (PGC) and formed the TS component of the TS and OCD Working Group of the PGC.

## The tourette international collaborative genetics (TIC genetics) study—whole exome sequencing in families with TS

The TIC Genetics Study is a large, multi-center effort established in 2011 (http://tic-genetics.org) with several goals, including (1) to create a large, central repository for sharing clinical data and biomaterials from genotypically and phenotypically well-characterized affected individuals and their relatives; (2) to increase our understanding of the genetic architecture of tic disorders through identification of risk genes and loci, and enumeration of the number of these genes and loci that contribute risk; and (3) to leverage these findings alongside systems biological approaches to provide insights into the neurobiology underlying these disorders (Dietrich et al., 2014).

Patients are recruited at more than 20 sites from USA, Europe, and South Korea, including academic research and mental health care centers (Dietrich et al., 2014). Recruiting focusses on both multiplex families and apparently-simplex trios. Following extensive phenotyping, blood is drawn and processed at the NIMH Center for Collaborative Genomics Research on Mental Disorders at RUCDR (http://www.rucdr.org) for DNA and RNA extraction, lymphocytes cryopreservation, and lymphoblastoid cell lines establishment. Anonymized clinical data and biomaterials are stored in a sharing repository located within the National Institute for Mental Health Center for Collaborative Genomics Research on Mental Disorders (www.nimhgenetics.org). Importantly, this study has been designed to optimize compatibility with other TS genetic consortia and researchers, as this will be critical to advancing our understanding of this disorder (Dietrich et al., 2014).

The TIC Genetics study leverages multiple genomewide approaches for identifying rare, large effect size variants, focusing both on identifying highly penetrant genetic variants segregating in multiply affected pedigrees and on *de novo* mutations identified in simplex families. Genomewide methods include genotyping microarrays for linkage analysis (Ercan-Sencicek et al., 2010) and CNV detection (Fernandez et al., 2012), and whole-exome sequencing (WES) for SNP and insertion-deletion variant (indel) detection. Efforts of TIC Genetics investigators led to the implication of histaminergic pathway genes in TS etiology (Ercan-Sencicek et al., 2010; Fernandez et al., 2012).

TIC Genetics is currently finishing analysis of WES data from 325 simplex TS trios. The main focus is the detection of *de novo* SNPs and indels, largely due to the success of this gene discovery approach in ASD (Sanders et al., 2015). Because of the rarity of *de novo* mutations and their large effect size, recurrent mutations can be leveraged to identify risk genes with high confidence. Excitingly, the identification of risk genes, in a hypothesis-free manner, facilitates systems biological analyses aimed at answering critical questions about the underlying neurobiology of TS. Systems approaches alongside gene-expression data from the developing human brain may have already been quite fruitful in this regard (Willsey et al., 2013).

## EMTICS: european multicentre tics in children study; exploring gene-environment interactions that underlie TS etiology

EMTICS is a multi-national study funded by the European Commission under the Seventh Framework Programme, including 17 clinical sites from across Europe (http://emtics.eu). It is prospectively designed to offer, for the first time, the opportunity to evaluate environmental risk factors that may lead to tic exacerbation but also, new tic onset, while correlating to genomic background. Two unique patient cohorts form the core of EMTICS: The ONSET study involves follow-up of 375 high-risk children aged 3–10 years who have a first degree relative with a diagnosis of TS and at study entry have no tics. The COURSE study includes and follows for up to 3 years, 700 children, and adolescents aged 3–16 years with a known chronic tic disorder or TS.

Individual genetic background alone cannot predict the risk for TS and a role of exposure to psychosocial stress, pre- and perinatal difficulties, and GAS infections (Hoekstra et al., 2013; Mathews et al., 2014) in TS etiology has been shown. The human pathogen GAS is a major cause of common pharyngitis, but also of significant post-streptococcal autoimmune multi-organ sequelae associated with the existence of host autoantibodies against GAS antigens, including rheumatic fever and Sydenham's chorea (Church et al., 2002). In the 1990s, Swedo et al. (1998) described a clinical phenotype, named Paediatric Autoimmune Neuropsychiatric Disorders Associated with Streptococcal Infections (PANDAS). Although it is still controversial whether PANDAS criteria can be used to designate a unique clinical entity, further research into the potential role of the innate and adaptive immune systems in the pathogenesis of tics and OCD is warranted (Martino et al., 2009; Murphy et al., 2010). The etiological link between GAS infections and TS/OCD may be related to an autoimmune process, following the model of molecular mimicry, according to which structural similarity between streptococcal and cerebral antigens might elicit a pathogenic cross-reactivity of antibodies originally targeting GAS antigens to host antigens.

Within EMTICS, the main hypothesis is that the onset of TS is dependent on identifiable genetic factors interacting with identifiable environmental factors. The study aims to test the likelihood that the development of new tics or tic exacerbation in individuals with a specific genetic background, is increased by recent exposure to pharyngeal GAS carriage or infection. The study investigates the genomic background of studied individuals through genomewide genotyping, in relation to new tic onset and tic exacerbation, correlating to the presence of GAS in throat swabs, comorbidities, pre- and perinatal difficulties, psychosocial stress (also measured via cortisol levels in hair follicles), and immunological measures. Transcriptome-wide gene-expression profiles of patients at points of tic exacerbation and tic remission as well as before and after tic onset in newly-diagnosed patients will also reveal, for the first time, pathways that are activated during the course or onset of TS. The first patient was enrolled in March 2013 and the study will conclude in 2017. The potential observation of a pathogenic link between an environmental immune-activating factor and risk for the development of a tic disorder and/or OCD may pave the way to the application of immune-modulating prophylactic and treatment approaches in these conditions.

## TS-EUROTRAIN—coordinating large-scale studies and training the next generation of experts for TS

In an effort to address the need for large-scale collaboration in order to tackle the multi-faceted etiology of TS but also train the next generation of young experts in the field, the Marie Curie Initial Training Network TS-EUROTRAIN was established, supported by the European Commission (http://ts-eurotrain.eu). Collaborative efforts of 14 academic institutes along with 12 PhD students form a highly multidisciplinary and inter-sectorial team, with the European experts in the study of TS collaborating with leading scientists in the USA. Building bridges between academia and industry is key to the network with two industrial partners providing pioneering expertise to the network: deCODE Genetics, a large genetic services and research provider and Boehringer Ingelheim PHARMA, one of the 20 largest pharmaceutical companies in the world. Twelve individual, yet complementary, projects interact to form a comprehensive study of TS and comorbidities from genetics and epigenetics through to physiology, brain anatomy, and function. These projects can roughly be divided into three groups by their main approach; genetic (and epigenetic), animal models, and human neuroimaging, respectively.

TS-EUROTRAIN aspires to act as an interface bringing together multiple large-scale efforts in the field. The main scientific goals are to assemble and interrogate a large genetic database for the evaluation of the genetic architecture of TS, to explore the role of gene-environment interactions in TS etiology including, for the first time, the effects of epigenetic phenomena, and to gain new insights into the neurobiological mechanisms of TS via cross-sectional and longitudinal neuroimaging studies and animal studies. Among the main expected outcomes of TS-EUROTRAIN will be the largest meta-analysis of European patient cohorts, resulting in a total of about 3000 patients with TS analysed for about 700,000 genetic markers across the genome as well as genomewide CNV studies in European patient cohorts. Furthermore, already, TS-EUROTRAIN has produced the first ever epigenome-wide association study for tics, analysing data from the Netherlands Twin Register (Zilhão et al., 2015). This study interrogated 411,469 autosomal methylation sites in 1678 individuals. Although no site reached genomewide significance, the top hits include several genes and regions previously associated with neurological disorders and warrant further investigation (Zilhão et al., 2015). Systems biology approaches and integration of data from multiple sources are main aspects of TS-EUROTRAIN methodology. Thus, large-scale data analysis and novel algorithm development for integration of data from “omics” platforms but also clinical and neuroimaging data are important parts of the study.

The academic and industrial partners form a unified training infrastructure to provide interdisciplinary training for TS. Specialized training covers cutting-edge scientific areas ranging from basic neuroscience and genomics to bioinformatics and computer science. Direct interaction of the network with European patient groups (Tourette-Gesellschaft Deutschland e.V., Germany and Netherlands Foundation of patients with TS, Netherlands) provides a unique opportunity to learn from patients and disseminate scientific knowledge of TS to large non-scientific audiences. Undertaking a comprehensive scientific and outreach programme, TS-EUROTRAIN aims to build Pan-European infrastructure and render TS into an example disorder for the study of other neurodevelopmental disorders and the development of European policies for the promotion of childhood mental health.

## Conclusions

Collaborative efforts of dedicated researchers from around the world have brought us on the verge of a new era, promising exciting, and rapid discoveries in the field of TS genetics. Multiple resources are coming together for TS genetic research; large well-characterized patient cohorts, specialized epidemiological databases, novel genomics technologies, and sophisticated methodology for the analysis of large-scale datasets. Systems biology approaches and integration of data from multiple sources and “omics” platforms can be expected to reveal novel facets of TS etiology, while cross-disorder meta-analysis for the identification of overlapping risk factors is shifting our view toward a whole spectrum of neurodevelopmental phenotypes. Importantly, the individual large-scale efforts described here, are ultimately joining their powers with the goal to boost power and identify definitive susceptibility genes for TS. These scientific alliances in concert with parallel large scale efforts in psychiatric genetics such as the PGC hold the promise to get us over the “precipice” and enter a new phase in TS gene discovery that may lead us to new pathophysiologic mechanisms underlying the disorder.

## Author contributions

All authors listed, have made substantial, direct and intellectual contribution to the work, and approved it for publication.

## Funding

EMTICS and TS-EUROTRAIN are funded by the European Union under the Seventh Framework People Programme (TS-EUROTRAIN (FP7-PEOPLE-2012-ITN), Grant Agr.No.316978—EMTICS (FP7/2007-2013), Grant Agr.No. 278367). TSAICG is funded by NIH U01NS40024, U01NS40024-09S1, K23MH085057, R01MH096767, K02NS085048, and research funding from the Tourette Association of America. TIC Genetics is funded by NIH (R01MH092290; R01MH092291; R01MH092292; R01MH092293; R01MH092513; R01MH092516; R01MH092520; R01MH092289; U24MH068457) and the New Jersey Center for Tourette Syndrome and Associated Disorders (NJCTS).

### Conflict of interest statement

JS and CM have received grant and travel support from the Tourette Association of America. The other authors declare that the research was conducted in the absence of any commercial or financial relationships that could be construed as a potential conflict of interest.
